# Exploring Gender Differences in Social Support in Cardiac Rehabilitation: A Comprehensive Review

**DOI:** 10.7759/cureus.89333

**Published:** 2025-08-04

**Authors:** Laura E Marchwinski

**Affiliations:** 1 Human Movement Science, Oakland University, Rochester, USA

**Keywords:** cardiac rehabilitation (cr), gender-differences, phase 2 cardiac rehabilitation (cr2), program completion, social support intervention

## Abstract

This comprehensive narrative review examines how social support influences participation in Phase 2 cardiac rehabilitation (CR2), a commonly structured 12-week outpatient program focused on supervised exercise, education, and lifestyle change. Literature published between 2005 and the present was reviewed across four databases, resulting in four studies that met the inclusion criteria. Social support was described across three dimensions (emotional, informational, and practical) to explore how these forms of support intersect with gender‑related patterns in health behaviors, coping, and engagement in CR2. Key findings indicated that women tend to rely more on emotional support, while men often benefit from practical assistance. Non‑traditional support networks, such as neighbors and volunteers, were also found to facilitate participation for both genders. These insights highlight the need for gender‑sensitive CR2 programming and policies to strengthen support systems, address disparities in involvement, and improve long‑term recovery outcomes.

## Introduction and background

Overview of heart disease and cardiac rehabilitation

Heart disease is the leading cause of death in the United States, accounting for 919,032 deaths in 2023. This represents roughly one in every three deaths nationwide and affects people across nearly all racial and ethnic groups [1]. These complications often require medical interventions such as angioplasty, coronary bypass surgery, stent placement, or even heart transplantation. Following these qualifying cardiac events, individuals are often referred to cardiac rehabilitation (CR), a comprehensive program designed to aid their recovery [2].

The importance of cardiac rehabilitation phase 2 (CR2)

CR is typically divided into three phases. Phase 1 begins in the hospital setting immediately following a cardiac event, focusing on early mobilization and education. Phase 2 (CR2), the focus of this review, is most commonly structured as a 12-week outpatient program involving supervised exercise three times per week, ongoing education, and lifestyle modification support. While this model is widely adopted, program length and frequency may vary by institution, country, and insurer. Phase 3 then emphasizes long-term maintenance and continuation of health behaviors. CR2 has shown numerous benefits, including improved cardiovascular fitness, reduced risk of future cardiac events, enhanced quality of life, and improved psychological well-being [2].

Gender disparities in CR2 participation

Despite CR2’s clear benefits, participation rates remain low, particularly among women [3]. Fewer than 30% of eligible individuals engage in CR2 programs [3], and women are 9-13% less likely than men to enroll and complete CR2 [4]. This disparity has been linked to caregiving responsibilities, transportation barriers, cultural expectations, and lower referral rates from providers [4]. One key factor influencing both participation and adherence is social support, which encompasses emotional, informational, and practical assistance from family, friends, and healthcare providers [5].

Social Support Theory

This review was guided by Social Support Theory, which served as the conceptual framework for categorizing and interpreting findings. It was selected because it provides a well‑established model for understanding emotional, informational, and practical support and how these forms of support influence health behaviors [5], including CR2 participation. Social Support Theory proposes that individuals who perceive strong support networks are better able to cope with challenges, manage stress, and maintain overall well‑being [5]. Emotional support offers comfort and reassurance, informational support provides knowledge and guidance, and practical support assists with daily tasks and logistics [5]. Together, these dimensions buffer stress, reduce the negative effects of illness, and increase adherence to health programs like CR2.

In this context, Social Support Theory underscores that strong support systems can enhance participation, decrease feelings of isolation, and promote better psychological and physical recovery. While this review primarily focused on gender differences, several included studies also addressed intersecting factors such as age, ethnicity, and socioeconomic status, which can shape how support is accessed and experienced.

Dimensions of social support

Emotional support refers to the expressions of empathy, love, trust, and caring that individuals receive from their social network [5,6]. This support often comes from listening, comforting, and encouraging [5,6]. For example, a spouse offering reassurance or a close friend providing a listening ear can deliver the emotional stability needed to navigate the challenges of rehabilitation.

Informational support involves providing advice, guidance, and knowledge to help individuals make informed decisions about their health [5,6]. In the context of CR2, this could include medical advice from healthcare providers, educational materials about lifestyle changes, or insights from peers who have had similar experiences. Informational support is essential for helping patients understand their condition and the importance of adhering to program protocols, such as exercise regimens and dietary recommendations [6].

Practical support includes tangible help with everyday tasks, such as transportation to and from CR2 sessions or financial assistance. For many individuals, practical support can significantly reduce barriers to participation, especially for those with physical limitations after a cardiac event [6]. This dimension of support may ensure that individuals can regularly attend CR2 sessions and continue in the program without unnecessary setbacks [6].

According to Social Support Theory, the emotional, informational, and practical dimensions of support work together to buffer the adverse effects of stress, enhance overall well‑being, and ultimately promote adherence to health behaviors [5]. These dimensions of support help individuals navigate the challenges of rehabilitation, improve their coping mechanisms, and reduce feelings of isolation, all of which are essential for sustaining participation and achieving better recovery outcomes in CR2 programs [5]. Social Support Theory emphasizes not just the presence of support but also its quality in helping individuals meet the demands of recovery and successfully navigate the rehabilitation process. This review considers both perceived and actual support, as perception often influences willingness to participate, while tangible assistance helps overcome logistical barriers to CR2 attendance.

Purpose

Understanding the role of social support is essential for addressing barriers to participation and ensuring that all individuals can benefit from CR2 [6]. Gender differences in how social support is perceived and utilized during CR2 may help explain why certain groups, particularly women, have lower engagement rates [7]. Examining these dynamics is important for improving patient outcomes and ensuring equitable access to CR programs [8].

This comprehensive narrative review synthesizes literature published from 2005 to the present, examining how social support influences CR2 participation, with attention to gender differences. By mapping the evidence and identifying common themes, this review highlights gaps in knowledge and provides insights to inform gender‑sensitive CR2 programming and policy.

## Review

Methods

This comprehensive narrative review examined the role of social support in Phase 2 cardiac rehabilitation (CR2) and explored gender differences in how support is experienced and utilized. A systematic search was performed across four databases: Google Scholar, PubMed, MedlinePlus, and the Library OneSearch tool. The search covered publications from 2005 to the present and used the Boolean string (“cardiac rehabilitation” OR “CR2”) AND (“social support”) AND (“gender differences” OR “sex differences”) AND (“participation rates” OR “program adherence”). The search identified 484 citations: 355 from Google Scholar, 129 from Library OneSearch, and none from PubMed or MedlinePlus. No unique studies were retrieved from PubMed or MedlinePlus; the citations found there were either duplicates already captured in other databases or focused on topics outside the scope of CR2.

To ensure transparency, the inclusion and exclusion criteria were defined at the outset. Studies were included if they were peer‑reviewed, published in English between 2005 and the present, examined adult populations, and addressed social support in CR2 or analogous rehabilitation programs, with attention to gender differences. Studies were excluded if they were pediatric, non‑English, lacked a focus on social support, or centered on unrelated health behaviors. Because very few studies addressed gender differences in social support specific to CR2, the inclusion criteria were carefully broadened to encompass literature examining gender‑based differences in social support in other chronic illness and health behavior contexts. This expansion provided conceptual depth and comparative insight without shifting the review’s primary focus on CR2.

After removing duplicates, 460 unique records remained for screening. Titles and abstracts were reviewed for relevance, followed by full‑text assessment of potentially eligible studies. Four studies ultimately met the inclusion criteria. All screening and data extraction were conducted by the author. A structured matrix was used to extract study design, population, sample size, setting, social support dimensions examined, and key findings. Because this review was conducted by a single author, interrater reliability was not applicable.

**Figure 1:**
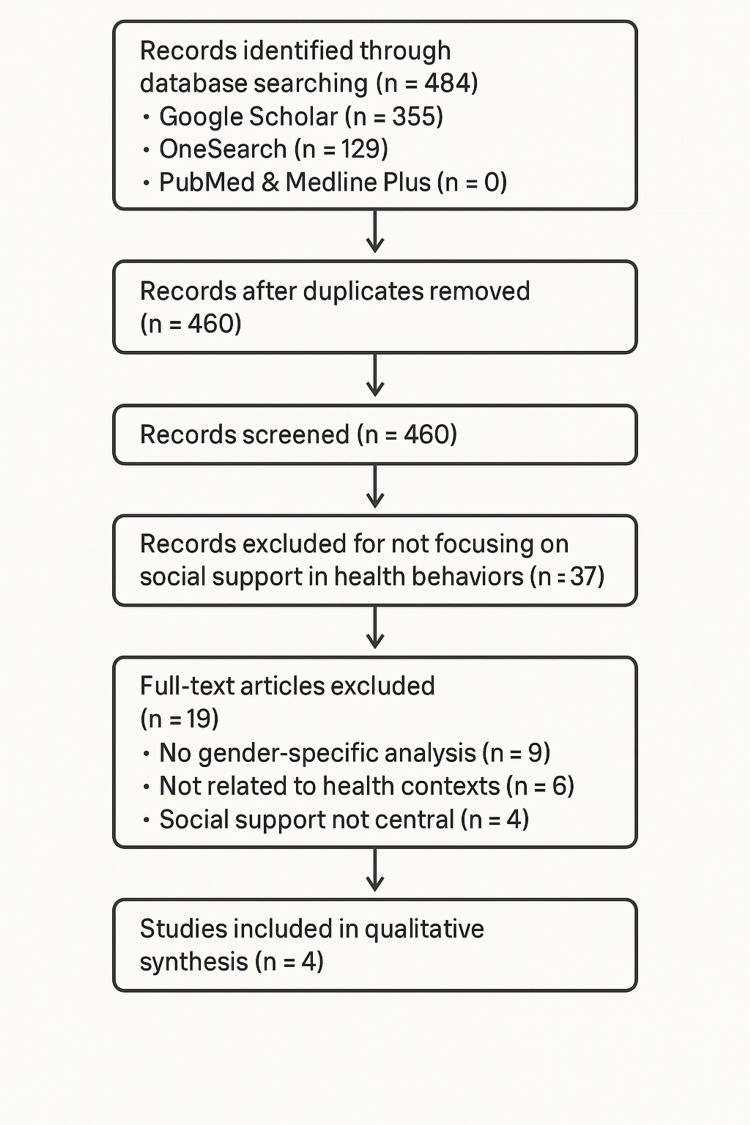
Flow diagram of study selection process. Relevant data were extracted from the selected studies, with a focus on social support dimensions (emotional, informational, and practical), gender-specific experiences, and participation outcomes in CR2 or analogous rehabilitation settings. The findings were analyzed using Social Support Theory as a guiding framework to identify common themes, highlight differences across studies, and reveal gaps in literature. The analysis informed conclusions about the impact of gendered experiences of social support on CR2 participation, emphasizing the need for gender-sensitive strategies in future program design and research.

A formal risk-of-bias or quality appraisal was not performed because of the small number and heterogeneity of included studies. Instead, findings were integrated using a narrative synthesis approach. Data were grouped according to the three dimensions of emotional, informational, and practical support outlined by Social Support Theory. The four included studies represented a range of designs (observational, cohort, and meta‑analysis) and sample sizes, from small qualitative studies to large cohorts involving more than 6,000 participants, providing a diverse foundation for identifying key themes and knowledge gaps (see Figure 1).

Literature review

Role of Social Support in CR2 Participation

Social support encompasses various forms of assistance that individuals receive from their social networks [5,9]. It has been well established that social support is significant in promoting adherence to health programs and improving health outcomes [7,9,10,11]. Studies consistently demonstrate that individuals with strong social support systems are more likely to participate in rehabilitation programs and experience better recovery trajectories [9,10]. However, the nuances of how social support functions in CR2, particularly regarding gender differences, remain less understood.

Social support is an integral aspect of health and well‑being, defined by Langford et al. as the perceived comfort, care, assistance, and information individuals receive from their social networks [5]. Research has consistently highlighted the role of social support in health outcomes, particularly for individuals facing chronic health conditions such as cardiovascular diseases [5]. In the context of CR2, social support not only facilitates adherence to treatment protocols but also enhances emotional well‑being, reducing the risk of depression and anxiety during recovery [5,9].

Recent research, including a quantitative cohort study by Matthews et al., illustrates the importance of social support in CR2 [9]. Matthews et al. examined 1,253 Medicare beneficiaries aged 65 and older from the ARIC (Atherosclerosis Risk in Communities) study [9]. Social support was measured with the Interpersonal Support Evaluation List-Short Form and social isolation with the Lubben Social Network Scale [9]. The study defined CR2 participation as attending at least one session based on Medicare claims [9]. Findings showed that higher social support was linearly associated with greater CR2 participation, while social isolation had a nonsignificant inverted J‑shaped relationship [9]. Participation rates overall were low, with only 23% of eligible older adults engaging in CR2, and there were no significant differences by race, sex, or age in how social support influenced participation [9]. This study underscores the importance of evaluating the quality and quantity of social support for older adults, though it also notes limitations, such as the absence of referral data and the lag between support measurement and CR participation (median 12.1 years) [9].

Gender Differences in Social Support Experiences

Research has shown that gender differences significantly influence stress perceptions and coping strategies, shaping how individuals utilize social support [12]. A quantitative internet survey by Kneavel et al. of 1,080 adults assessed the quality and quantity of social support alongside gender, age, and perceived stress [12]. The study found that women reported higher levels of stress, broader support networks, and greater perceived quality of social support, while men reported narrower networks and more task‑oriented coping [12]. The relationship between support quality and stress was curvilinear and moderated by gender, suggesting that for women, social support can serve both as a coping mechanism and, in some cases, as a source of stress [12]. Although this study was not CR2‑specific, it provides critical context for understanding how gendered stress and support‑seeking patterns may affect CR2 engagement.

Beyond coping styles, gender differences also influence health behaviors, access to social support, and management of chronic disease. Men may be less likely to seek help or express vulnerability due to societal expectations surrounding masculinity, while women often rely on relational connections and emotional sharing [12]. These findings were based on survey and thematic data from included studies, rather than implying direct causality. Women’s access to support may also be constrained by caregiving roles and responsibilities, which can reduce their ability to attend CR2 sessions. Together, these gendered factors, combined with intersectional influences such as age, race, and socioeconomic status, highlight the need for targeted strategies that recognize how different groups experience and utilize social support.

Impact of Gender on CR2 Participation

Research indicates that men and women may experience and utilize social support differently, particularly within the context of CR2 [6]. While both genders require social support, their specific needs and the availability versus effectiveness of support can vary. For example, men may benefit more from direct, task‑oriented, or logistical support, whereas women often seek emotional reassurance and connection [6,7]. Studies exploring the relationship between social anxiety, communication styles, and perceived social support show that gender influences these dynamics [5,9,12,13]. Validated survey instruments found that men scored higher in verbal aggressiveness, while women were more likely to demonstrate emotional expressiveness [7]. Most of these findings derive from Western, urban populations, and cultural differences in coping strategies may influence how widely these patterns generalize.

A peer‑reviewed observational study by Deb et al. examined 844 CR2 patients (607 men and 237 women) at the University of Michigan between 2017 and 2019 [6]. “Graduation” was defined as completing at least 75% of prescribed CR2 sessions. Participants rated their perceived social support on a 0-10 scale (0-6 low support; 7-10 high) [6]. Both men and women with high support had significantly higher graduation rates (p<0.01) [6]. For men, support from family and friends (a category that included spouses) most strongly influenced graduation (p<0.01), while for both genders, non‑traditional support networks, including neighbors and structured volunteer programs, were also linked to higher completion (each p<0.01) [6]. Notably, marital status and relationship quality were not associated with completion for either gender [6]. Because the study was observational, causality cannot be assumed, but the findings highlight the important role of diverse, non‑spousal support systems.

Deb’s findings can be contrasted with those of Molloy et al., who conducted a meta‑regression of 11 studies including 6,984 CHD patients [14]. They found that being married or partnered was associated with 1.72 times higher odds of CR attendance (95% CI: 1.50-1.97), with no evidence of heterogeneity or publication bias [14]. This suggests marital/partner support provides substantial emotional and practical assistance for CR participation. However, timeframe and methodology differences (meta‑regression from 2008 vs. a single‑site study from 2020) may help explain why Deb et al. did not find marital status to affect CR2 completion.

Taken together, these findings underscore the complexity of social support in CR2. Programs should consider both traditional networks (e.g., spouses) and non‑traditional supports (e.g., peers, volunteers, neighbors, healthcare providers) when designing interventions. For some men, provider encouragement and practical support may serve as a key motivator; for women, emotional reassurance and relational support may matter more. These nuances, along with intersectional factors like age, SES, and culture, highlight the need for gender‑sensitive and inclusive CR2 strategies. A summary of the articles reviewed can be seen in Table 1.

**Table 1:** Summary of Studies on Gender Differences and Social Support in Cardiac Rehabilitation This table summarizes four key studies included in the review focusing on social support and gender differences in cardiac rehabilitation phase 2 (CR2). CR2 is a 12-week outpatient program emphasizing exercise and education. Social support dimensions include emotional (empathy, reassurance), informational (advice, guidance), and practical (tangible assistance). The studies employ various research methods such as observational studies, survey-based research, and meta-regression analysis. Reference citations for these studies are provided in the main text. CHD: coronary heart disease.

Study (Author, Year)	Sample/Setting	Social Support Dimensions	Key Findings
Deb et al., 2020 [6]	844 participants; University of Michigan; CR2 setting	Emotional, Informational, Practical	High social support linked to higher CR2 completion; men benefited from family/friends; non-traditional networks (neighbors, volunteers) significant for both genders
Matthews et al., 2024 [9]	Older adults, Medicare beneficiaries	Emotional, Practical	Increased social support associated with higher CR2 participation; social isolation reduced engagement
Kneavel et al., 2021 [12]	Mixed gender chronic illness patients	Emotional, Practical	Men use task-oriented coping relying on practical support; women seek emotional reassurance, influencing CR2 engagement
Molloy et al., 2008 [14]	Meta-regression, 6,984 CHD patients	Emotional, Practical	Married/partnered individuals 72% more likely to attend CR2, highlighting importance of marital support

Discussion

The insights gained from understanding social support and gender differences have important implications for how CR2 programs are designed and delivered. Communication skills training could help participants more effectively express their needs to family, friends, and care providers. For example, short role‑play sessions or group discussions could teach women to feel comfortable requesting emotional reassurance and men to confidently seek practical assistance, such as transportation or scheduling help. Providers can involve families without overburdening them by inviting relatives to select education sessions or milestone updates, offering options for virtual attendance when needed. Clear distinctions between short‑term outcomes, such as immediate participation and adherence during CR2, and long‑term outcomes, such as sustained exercise habits and reduced hospitalizations, are essential. Most current research stops short of measuring what happens after the program ends. More longitudinal studies are needed to determine whether gender‑sensitive strategies have lasting effects.

Concrete examples of gender‑sensitive interventions include women’s peer support groups, logistical support programs for men, and hybrid in‑person and digital mentorship models. Programs could assess support quality using standardized tools and explore approaches such as gender‑targeted peer mentors or respite care resources for participants with caregiving responsibilities. Cultural and socioeconomic factors also influence how support is accessed, requiring flexibility in program design. For example, transportation subsidies may be most important for lower‑income patients, while community volunteer networks could assist in rural areas. Digital supports, such as telehealth check‑ins, online discussion forums, and text reminders, could expand reach and help overcome barriers for women balancing caregiving or work schedules.

This review used a narrative approach guided by Social Support Theory but did not include a formal quality assessment, which is a limitation that underscores the need for more rigorous research and evaluation of emerging gender‑sensitive CR2 interventions.

## Conclusions

Social support plays a vital role in participation and success during CR2, influencing how individuals engage with programs and recover. Recognizing gender differences in support needs helps remove barriers, strengthen connections, and enhance overall outcomes for participants.

By incorporating gender‑sensitive approaches, CR2 programs can develop inclusive and effective support systems that extend beyond traditional family structures, integrate digital and community resources, and address the real‑world challenges faced by both men and women. These strategies can promote greater participation, support sustained recovery, and ultimately improve the quality of life for individuals rebuilding their health after a cardiac event.
